# Influence of ZnSO_4_ and Methyl Jasmonate on the Metabolites and Bioactivity Present in Lemon-Fruit Membrane Vesicles

**DOI:** 10.3390/ijms252312917

**Published:** 2024-11-30

**Authors:** Maria Gomez-Molina, Micaela Carvajal, Paula Garcia-Ibañez

**Affiliations:** Group of Aquaporins, Department of Plant Nutrition, Centro de Edafología y Biología Aplicada del Segura (CEBAS-CSIC), Campus Universitario de Espinardo, Apdo. de Correos 4195, 30080 Murcia, Spain

**Keywords:** *Citrus*, nanovesicles, phenolics, biostimulation, sustainability

## Abstract

Membrane vesicles isolated from vegetable tissues have shown promise in encapsulation technologies used in industries like food and cosmetics, offering innovative approaches to product development. However, their associated linked metabolites have not been studied. Lemon vesicle research not only adds value to the lemon crop (*Citrus × limon* L.), one of the most widely cultivated fruit trees in the world, is a source of bioactive compounds such as phenolics and organic acids. In this study, the influence of elicitation with ZnSO_4_ and methyl jasmonate, which modulate the bioactive metabolites, on fruit membrane vesicle bond metabolites was studied. The study showed that foliar application of ZnSO_4_ increased phenolic compounds as caffeic, sinapic, and chlorogenic acids and the flavonoid hesperidin by about 20% in lemons. Furthermore, a clear interaction between vesicles and citrate and ascorbate that were increased by methyl jasmonate were associated with their higher bioactivity. This was related to the double intrinsic in vitro antioxidant activities of these vesicles.

## 1. Introduction

Lemon fruits (*Citrus × limon* L.) are among the most widely cultivated and economically significant fruit crops in the world. In particular, they play a crucial role in the Spanish market, with production reaching 857,000 tons in 2022. One of the primary producers is the region of Murcia, accounting for over 60% of the total output [1] As lemons are a source of high bioactive compounds [2], processes of re-valorization and their strategic transformation could not only result in the production of high-value products with applications in various biotechnological fields such as nutraceuticals or cosmetics, but also contribute to a reduction in the environmental impact and the fostering of the circular economy [3,4]. 

In response to the search for new industrial applications, plant-derived membrane vesicles have been identified as a versatile nanocarrier due to their unique properties. Their biocompatibility, high stability, and availability enable them to penetrate biological membranes with high performance and minimal immunological response, rendering them highly interesting in the cosmetic and pharmaceutical industries [5,6]. Moreover, their ability to withstand the conditions of the gastrointestinal tract has also increased their interest in the food industry. Furthermore, their potential in the nutraceutical or food industry is based on their capacity to enhance stability through the digestive tract, surviving the acidic pH of the stomach [7]. With regard to lemon-derived membrane vesicles, in vitro studies have indicated the potential for these vesicles to exhibit anticancer, anti-inflammatory, and antioxidant properties [8,9]. 

The general characterization of lemon membranes has been related to their specific molecular composition in phospholipids, sterols, and proteins [10]. A recent study conducted by Olmos-Ruiz et al. (2024) [11] provided a more comprehensive insight into the composition of citrus-derived vesicles, focusing on fatty acids and sterols and the proteomic analysis. Nevertheless, there is limited information currently available on how metabolites, such as phenolic compounds and organic acids, interact with membrane vesicles, which are a crucial factor in determining their bioactive activity. In citrus fruits, phenolic compounds primarily function as antioxidants not only through the donation of protons or electrons, but also by interacting with various enzymes involved in oxidation processes such as lipoxygenase [12]. The previously mentioned encompass a wide range of compounds including phenolic acids like caffeic acid and sinapic acid as well as flavanones such as naringenin and hesperidin or organic acids such as citrate, malate, or ascorbate. Understanding how these primary and secondary metabolites interact with membrane vesicles could provide valuable insights into their bioavailability and efficacy, highlighting their potential as functional ingredients in health and nutrition applications.

On the other hand, elicitation is defined as the process of enhancing or inducing the synthesis of metabolites including phenolic compounds in plants. In addition, the use of elicitation products in field production has increased over the last years due to their enhancement of primary and secondary metabolism, promoting plant adaptation to further stresses or improving the growth and development of the plant [13]. Consequently, elicitation can enhance the revaluation of waste products due to the greater concentration of bioactive compounds [14,15]. Among the numerous elicitors, zinc (Zn) salts have been identified as a particularly promising biostimulant. In particular, ZnSO_4_ has been shown to elicit secondary metabolism and enhance metabolite content, thereby promoting improved plant growth and an enhancement in fruit quality [16,17,18]. Similarly, some phytohormones, such as methyl jasmonate, have been shown to enhance primary and secondary metabolism including increasing the synthesis of phenolic compounds in citrus [19,20]. However, although the function of these phytohormones has been broadly described in different crops, their use in citrus trees has only been recently established, so the impact of these practices on the resulting membrane vesicles and their further interaction with metabolites [21,22]. 

In light of these considerations, this study aimed to evaluate the effects of elicitation with methyl jasmonate and ZnSO_4_ on membrane vesicles derived from *C. limon*, with particular focus on the phenolic compounds found to be associated with them and the chemical metabolic composition. By investigating how these elicitation treatments influence the phenolic content and chemical properties of the vesicles, this research seeks to uncover their potential as valuable ingredients in high-value industries.

## 2. Results

### 2.1. Lemon Fruit Characterization

#### 2.1.1. Morphological Characteristics

In order to test the quality of the initial material and determine the potential impact of elicitors on the fruits, a series of tests were conducted on the physiological (fruit fresh weight, length and volume) and quality (pH and °Brix on the juice) parameters of the samples from C (control), M (methyl jasmonate treated), and Z (ZnSO_4_ treated) lemon trees (Table 1). The results showed a significant increase in the fresh weight of M treated lemons (about 17%) when compared to the control fruits. In addition, a higher volume was also observed in the M treated lemons in comparison to the control samples (*p* < 0.05). However, no statistically significant differences were observed in the other determined parameters (length, pH, and °Brix) between the M treated lemons and the control. Regarding the Z treatment, no statistically significant differences were observed when compared against the control. 

#### 2.1.2. Chemical Characteristics

**Figure 1:**
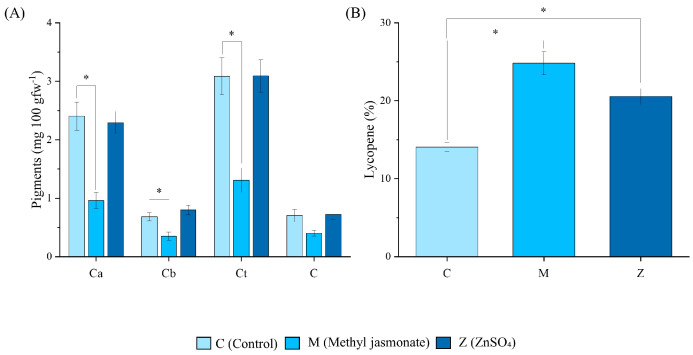
Pigments analysis of C (control), M (methyl jasmonate) and Z (ZnSO_4_) lemons (from lighter to darker blues, respectively). (**A**) Analysis of chlorophylls: chlorophyll a (Ca), chlorophyll b (Cb), total chlorophyll (Ct), and carotenoids (C). (**B**) Lycopene content of C, M, and Z lemons. Data represent the mean ± SEM (*n* = 3). The data were analyzed by a Student’s *t*-test between the treatment and the control. * *p* < 0.05.

The analysis of the content of chlorophyll a and b, carotenoids, (mg 100 gfw (grams of fresh weight)^−1^) (Figure 1A), and lycopene pigments (Figure 1B) (%) in the C (control), M (methyl jasmonate treated), and Z (ZnSO_4_ treated) lemon peel revealed that M lemons exhibited the lowest concentration of chlorophyll a, chlorophyll b, and total concentration when compared to the control treatment (*p* < 0.05). Although the concentration of carotenoids was similar, the lycopene content (as a carotenoid) was higher in the M lemons.

The total mineral content of macronutrients (Ca, K, Mg, P, and S) and micronutrients (B, Cu, Fe, Mn, Mo, Ni, and Zn) of the C (control), M (methyl jasmonate treated), and Z (ZnSO_4_ treated) lemons showed no statistically significant differences in macro- or micro-elements (Table S1).

**Figure 2:**
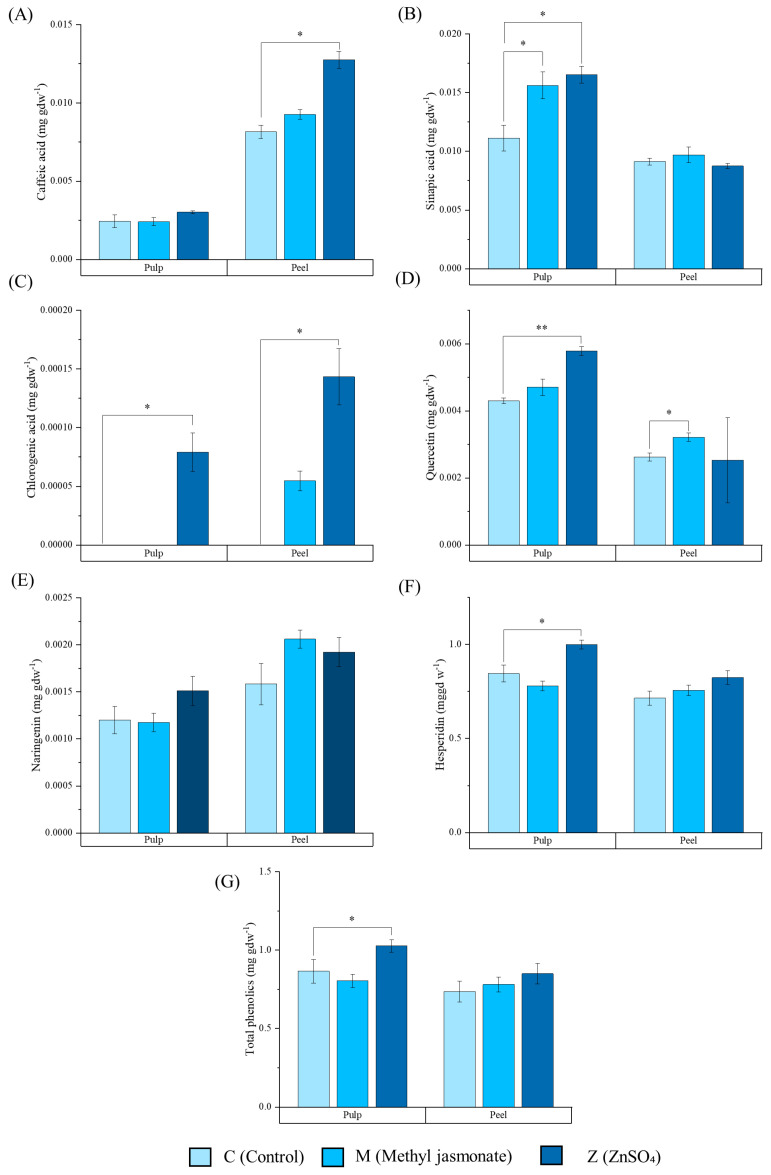
Phenolic content. (**A**) Caffeic acid, (**B**) sinapic acid, (**C**) chlorogenic acid, (**D**) quercetin, (**E**) naringenin, (**F**) hesperidin, and (**G**) total phenolics of the C (control), M (methyl jasmonate treated), and Z (ZnSO_4_ treated) (from lighter to darker blues, respectively) lemon pulps and peels in mg gdw^−1^ (grams of dry weight). Data represent the mean ± SEM (*n* = 3). The data were analyzed by a Student’s *t*-test between the treatment and the control. *p < 0.05. ** *p* < 0.01.

Pulp and peel from the C, M, and Z lemons showed significant differences according to its phenolic content (mg g.^−1^ d.w). According to the caffeic acid concentrations in the peel (Figure 2A), a statistically significant increase was only detected for the Z treatment when compared to the M treatment (*p* < 0.05). However, when analyzing the peel, an increase was observed with both treatments. With regard to sinapic acid (Figure 2B), only an increase in concentration after both M and Z treatments was observed in the lemon fruit pulp. As with chlorogenic acid (Figure 2C), it was only detected in the pulp of the Z treated lemons and in the M and Z treated lemon peels. Regarding the quercetin concentration (Figure 2D) in the lemon pulp, an increase was also observed by 25% after Z elicitation. Quercetin analysis in the peel revealed a 10% increase with the M treatment. Nevertheless, no statistically significant differences were obtained for the naringenin content (Figure 2E). Hesperidin concentrations (Figure 2F) demonstrated an increase by 15% with the Z treatment when compared to the C pulps. When regarding the whole phenolic content (Figure 2G), the total increase was observed for the Z treatment when compared to the C or M pulps, but no differences were observed in the total peel content.

### 2.2. Lemon Pulp Derived Membrane Vesicles Characterization 

#### 2.2.1. Size, Polydispersity, and Protein Yield

Following the study of the physicochemical and mineral composition, a study of the lemon-derived membrane vesicles was carried out. The vesicles isolated from the pulp of the C, M, and Z lemons respectively showed a protein yield of around 6.11 µg µL^−1^. Regarding the size of the vesicles, it was very similar among treatments, giving a mean value of about 678 nm for the vesicle size and a polydispersity index of 0.86, with no significant differences (*p* > 0.05) (Table 2). 

#### 2.2.2. Phenolic Content and Metabolites Associated with the Lemon Membrane Vesicles 

**Figure 3:**
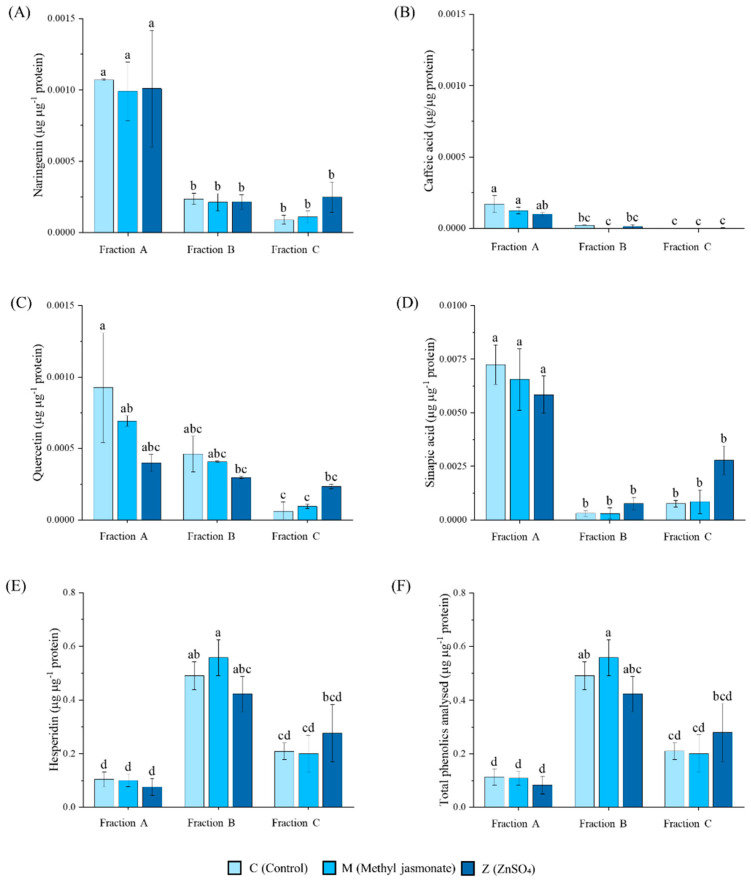
Phenolic content in (**A**) naringenin, (**B**) caffeic acid, (**C**) quercetin, (**D**) sinapic acid, (**E**) hesperidin, and (**F**) total of fractions A (polar), B (protein), and C (non-polar) of the C (control), M (methyl jasmonate treated), and Z (ZnSO_4_ treated) lemon pulp vesicles (from lighter to darker blues, respectively) in µg µg^−1^ protein. Data represent the mean ± SEM (*n* = 3). Different letters represent significant differences according to two-way ANOVA followed by Tukey’s post hoc test (*p* < 0.05).

Fractions A, B, and C of the C, M, and Z lemon vesicles showed significant differences (*p* < 0.05) over their phenolic content. Naringenin was predominantly present in fraction A of the C, M and Z vesicles, with no significant differences between treatments (*p* > 0.05) (Figure 3A). Additionally, caffeic acid was predominantly present in fraction A of the C and M vesicles (Figure 3B). With regard to quercetin, similar concentrations were found in fraction A of each type, and fraction B of the C and M vesicles, with the lowest prevalence in fraction C (Figure 3C). Similarly, sinapic acid exhibited a prevalence in fraction A of each type of vesicle, which was greater than that observed in the other fractions (Figure 3D). However, hesperidin was the predominant compound in fraction B of each type of vesicle, followed by fraction C, and finally, fraction A. As hesperidin was the predominant phenolic compound analyzed and there were no significant differences between treatments (Figure 3E), the total phenolic content performed similarly, resulting in a greater concentration in fraction B, where hesperidin predominated (Figure 3F). Chlorogenic acid was not identified in the vesicles.

Subsequently, a non-directed metabolomic assay was conducted on fraction A from vesicles derived from the C, M, and Z lemons, revealing that the vesicles retained bioactive compounds, among which organic acids and amino acids were prominent in its soluble fraction. Fraction A highlighted the presence of ascorbic acid and citrate among the main metabolites (Table 3). In the M vesicles, concretely, the ascorbate and citrate content was 68% higher than in the C vesicles, while malate was significantly higher in the Z vesicles, exceptionally. On the other hand, the non-protein amino acid gamma-aminobutyric acid (GABA) content was found to be around 69% higher than those in C; most protein amino acids such as alanine, asparagine, aspartate, glutamate, glutamine, proline, and valine were 2–4 times higher (*p* < 0.05).

### 2.3. Bioactivity Assays of the Lemon Membrane Vesicles

**Figure 4:**
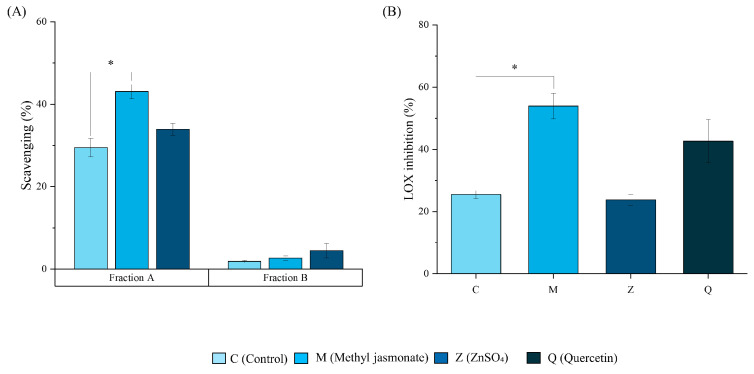
Antioxidant properties of nanovesicles. (**A**) Antioxidant activity (scavenging, %) of fractions A and B of the C (control), M (methyl jasmonate), and Z (ZnSO_4_) vesicles (from lighter to darker blues, respectively). (**B**) Antioxidant activity (LOX inhibition %) of fraction A of the C (control), M (methyl jasmonate), and Z (ZnSO_4_) vesicles using Q (quercetin 10 µg mL^−1^) as a positive control. Data represent the mean ± SEM (*n* = 4). The data were analyzed by a Student’s *t*-test between the treatment and the control. * *p* < 0.05.

The different experiments performed to understand the antioxidant potential of the lemon-derived membrane vesicles are illustrated in Figure 4. First, the radical scavenging activity of fraction A and fraction B was analyzed (Figure 4A). For fraction A, a statistically significant increase of 80% in scavenging activity was observed in the M samples. However, no differences were obtained for the protein phase (Fraction B). In this way, the enzyme assays were performed with fraction A. Figure 4B shows the inhibition analysis of the LOX enzyme in comparison with a positive control, quercetin (Q). The samples revealed that the membrane vesicles derived from M lemons exhibited a twice higher LOX inhibitory potential than those in C lemons (*p* < 0.05). 

## 3. Discussion

### 3.1. Effect of the Elicitation in Lemon Fruits 

Assessing the fruit quality after an elicitation treatment is critical to understanding whether the effect on yield or biomass increase has a detrimental effect on secondary metabolism or nutrient accumulation in the plant. In our study, the evaluation of these parameters was not only fundamental to study the effect of elicitation with methyl jasmonate (M) or ZnSO_4_ (Z) in lemon plants, but also to understand the starting point for the extraction of the membrane vesicles. Regarding the mineral content, it was not altered by the elicitation, so it denotes a neutrality of the treatment at the mineral level of the fruit. Regarding the physiological parameters, M lemons were observed to be higher in fresh weight and volume, but not in length. In addition, the pigment color shift produced by the decrease in chlorophylls, accompanied by an increase in lycopene, is a common indicator of ripening in many fruits. Methyl jasmonate could accelerate the ripening process in lemons, which would explain the degradation of chlorophylls (responsible for the green color) and the increase in carotenoids, which usually give yellow or orange tones to ripe fruit [23]. Furthermore, this could be related to the improvement in the post-harvest storage of lemons, extending the shelf life and increasing the duration of their antioxidant potential [24,25]. Lemon fruits are also a source of phenolic compounds, with caffeic acid, chlorogenic acid, sinapic acid, and quercetin being the most important phenolic acids, and naringenin and hesperidin being the most important flavanones. In our experiment, ZnSO_4_ demonstrated a potential role in increasing the phenolic content, mainly in pulp (Figure 2), as previously reported in other studies [26,27]. More specifically, it can be observed that in the peel, the synthesis of acids derived from coumaric acids (mainly chlorogenic acid and caffeic acid) was increased, suggesting a stimulation of these pathways by ZnSO_4_. In addition, it has been reported that Zn improves the performance of the enzyme phenylalanine ammonia lyase, which is mainly responsible for the biotransformation of this amino acid to trans-cinnamic acid, thus initiating the route of phenolic acid synthesis [26]. Moreover, the same dynamics can be observed in the pulp, and additionally, the hesperidin content increased by around 20%, whose contribution enhanced the total phenolic content. The mechanisms by which the addition of this salt increases the hesperidin levels have not been elucidated yet, providing the opportunity for future research into the influence of Zn on flavonoid synthesis. Based on the information obtained from the field elicitation treatments, we underscore that while methyl jasmonate appears to accelerate ripening in lemons, as indicated by the changes in pigment composition, the Zn treatment was effective in enhancing the levels of key phenolic compounds. On the other hand, the effects of elicitation on organoleptic properties such as some volatiles or organic acids are of interest for future studies.

### 3.2. Lemon Pulp Membrane Vesicles 

Recently, research on lemon-derived membrane vesicles and how farming methods can affect their composition has been developed. The potential of these vesicles lies in their possible use as carriers and stabilizers in a diversity of areas, such as in food nutrition, cosmetics, or even in agronomy, as part of phytosanitary products. However, the specific nature of the metabolites that may interact with these lemon-derived vesicles and thereby contribute to their bioactivity remains undetermined. In order to deepen this characterization, a previous analysis of size, polydispersity, and protein yield was performed. None of these parameters were altered after both elicitor treatments, and the vesicle size was similar to previously described vesicles from citrus and brassica plants [8,11,27,28]. Furthermore, the protein yield was increased due to the reduction in the pH of the extraction buffer compared to that previously used [11]. This could indicate that an acidic pH similar to that of lemon cell membranes favors the extraction of lipoprotein vesicles [29].

Focusing on phenolic compounds, our results showed that naringenin predominantly resided in fraction A due to its flavanone nature and structural limitations, preventing penetration into the lipids, thereby remaining within the vesicular lumen [30]. Although quercetin and naringenin have similar structures, their interactions with membranes were observed to be different. Quercetin was found in both fractions A and B, and despite being less hydrophobic than naringenin, showed a stronger association with the protein components of membranes [31]. Sinapic acid exhibits a similar behavior to naringenin, despite having a similar structure to caffeic acid. However, the presence of three hydroxyl groups in the sinapic acid structure could explain its strong interaction with membranes, as these groups are known to act as hydrogen bond donors, interacting with the oxygen atoms on the phospholipid polar heads [32]. Caffeic acid also predominantly remained in fraction A due to its polar structure [30]. In contrast, hesperidin predominated in fraction B, demonstrating a strong interaction with the membrane lipoproteins, with a low prevalence in fraction A. All phenolic compounds analyzed prevailed in fraction A, with a low presence in the remaining fractions, as expected due to its polar nature, except for hesperidin, whose contribution resulted in a higher concentration of total phenolic compounds in fraction B [33]. It was also observed that there was no correlation between the increase in phenolic compounds in lemon pulp and the concentration of phenolic compounds in the pulp vesicles. These results open the door to future research on the interaction of these metabolites with vesicles by confocal microscopy (e.g., by fluorescence labelling). Once these interactions have been elucidated, the contribution of each of these compounds to antioxidant activities could be studied by priming the vesicles and carrying out antioxidation tests with the different fractions.

The study of the primary metabolites detected in the soluble fraction of the vesicles revealed the presence of amino acids and other components of the tricarboxylic acid (TCA) cycle. In brief, according to the organic acids, ascorbate resulted in an increase in the methyl jasmonate eliciting treatment, concordant with several studies of in vitro crops [34,35]. Furthermore, the fact that this molecule interacts with the vesicles, probably due to strong interactions with the polar heads of the membrane layers, makes them an excellent carrier because they contain vitamin C, a natural antioxidant that is widely used in cosmetics [36]. On the other hand, the increase in citric acid in M vesicles is in line with previous findings of elevated citric acid levels in citrus following elicitation with methyl jasmonate [16,37]. Previous studies have demonstrated that methyl jasmonate is able to accumulate citric acid by downregulating the gene expression and activity of some enzymes present in the tricarboxylic acid cycle such as aconitase or glutamate dehydrogenase [38]. It is also possible that modifications to the vesicles could facilitate greater interaction with the polar groups of citric acid. Furthermore, the accumulation mechanisms of organic acids, such as citric acid, can be enhanced by modifying channel transport through elicitation in vesicles [39]. Malate concentrations were also higher in Z-derived vesicles. Additionally, an increase in malate concentration was observed exclusively in vesicles derived from lemons treated with ZnSO_4_ [40]. The study by Sagardoy et al. (2011) [41] demonstrated that the external presence of zinc decreased the activity of the enzyme malate dehydrogenase, which is responsible for transforming malate into oxaloacetate. Consequently, the application of ZnSO_4_ in our study appears to have contributed to the accumulation of this metabolite and its accumulation in the vesicles. This accumulation may be further influenced by pH-driven ion gradients, enzymatic modifications, or the elicitor-induced upregulation of transporter activity, mechanisms that warrant future proteomic analyses to clarify their role in compound retention.

On the other hand, among the detected single amino acids, the presence of GABA by 1.5 times higher in the M than in the C samples stood out. The involvement of GABA in plant metabolism has been observed through its accumulation in tissues as a response against abiotic and biotic stress, although it has recently also been linked to fruit ripening [42]. Likewise, it has recently been reported that the exogenous application of methyl jasmonate in strawberries is able to promote GABA synthesis in the fruit. This could explain a higher accumulation of this amino acid with methyl jasmonate treatment, and therefore a higher concentration of this compound associated with vesicle extraction [41]. Regarding the remaining amino acids, increases such as that of alanine in the case of vesicles derived from the Z and M treatments could be due to changes in the phospholipid configuration following the elicitation treatments. This is supported by the fact that these amino acids, with less hydrophobic non-polar residues, were less adsorbed on the membranes compared to other amino acids like phenylalanine, which did not suffer any changes in our study [43]. Regarding the increase in asparagine, aspartate, glutamine, glutamic acid, and proline in vesicles treated with Z and M, this may be attributed to an enhancement of the TCA cycle in the initial citrus fruits. This also correlated with the higher concentrations of citric acid in those samples, thus indicating that a higher concentration of these metabolites had been retained through the extraction process [44,45]. 

The antioxidant activity, assessed with the DPPH method, of fractions A and B from C provided interesting results since fraction B, which was more enriched in hesperidin and phenolic compounds in general, was not the one with the highest antioxidant capacity, being fraction A or soluble, showing a higher antioxidant activity by the mechanism of free radical scavenging by polar metabolites. This suggests that hesperidin may complex or interact with proteins in a non-available form to develop its native antioxidant activity, although interactions have mainly been studied in animal vesicles [46]. Moreover, the fact that fraction A of the M vesicles had a higher antioxidant capacity than the C and Z vesicles, despite not having a significant increase in phenolic content compared to the others, its organic acid content suggests that the greater antioxidant action was due to ascorbate and mostly citrate. Therefore, elicitation with methyl jasmonate increased the inherent antioxidant power of the vesicles as carriers, making them suitable for use as preservatives in formulations [16,36,47,48] Due to this result, only fraction A was considered for the antioxidant assays with in vitro enzymes. With regard to the bioactivity of the lemon-derived membrane vesicles, LOX is a key enzyme responsible for catalyzing the conversion of arachidonic acid into pro-inflammatory leukotrienes, among other inflammatory and oxidative functions. These are recognized as significant mediators of various inflammatory processes [49]. This assay is therefore a valuable tool for the screening of antioxidant and anti-inflammatory ingredients. The results demonstrated that M vesicles exhibited considerably higher antioxidant activity (*p* < 0.05), with a 54% LOX inhibition, comparable to that of quercetin around IC50 (Figure 4B) [50] As previously stated, it is possible that citrate is the molecule responsible for these activities. Previous studies have investigated the LOX inhibitory activity of citrate complexes, which may explain the higher activity of M vesicles. The levels of quercetin, a natural inhibitor, in all three types of vesicles were similar, suggesting that this may not be a contributing factor to the observed differences in activity [51,52,53]. However, the higher concentration of amino acids in the M vesicles may contribute to a higher antioxidant activity and inhibition of LOX as well as other potential beneficial activities such as anti-inflammatory [54]. However, other compounds not analyzed, such as terpenoids or pigments like carotenoids, naturally present in lemon pulp, could be found in these vesicles and contribute to their bioactivities [55]. Thus, the effect of the vesicles may be due to the balance between the scavenging capacity of the phenolic compounds and the ability to interact with other enzymes of the organic acids. Furthermore, these antioxidant and potentially anti-inflammatory activities should be tested in cellular models, such as keratinocytes or macrophages, to study their scalability as potential antioxidant ingredients in high-value industries such as cosmetics, agricultural, or agri-food [28]. As a conclusion, our research suggests that vesicles derived from different plant sources may contain specific metabolites, opening new avenues to explore plant-specific transporters and optimize functional ingredients based on their unique properties.

## 4. Materials and Methods

### 4.1. Plant Material and Elicitation Treatments 

*Citrus × limon* L. (lemon) trees, Fino variety (over 15 years old), were selected from the experimental field set in Desamparados, Alicante (38°04′06.4″ N, 0°59′31.0″ W) belonging to Citrus G.B. During the course of the experiment, the plants were under Mediterranean climate conditions. Elicitation started in the second production time of the year, the first week of September 2023, applying the treatment once every 10 days until the last week of October 2023 (four applications in total), when the lemon fruits were recollected (a week after the last application). Elicitation treatments (spraying 2 L of solution per tree, using an elicitation backpack) were as follows: water as the control (C lemons), 100 µM methyl jasmonate (M lemons), and 500 µM ZnSO_4_ (Z lemons); these were all supplemented with 0.1% of surfactant. The selection of dosages was based on previous assays performed in the group and the concentration of surfactant was based on a previous patent (PCT/ES2019/070457). 

### 4.2. Physiological Characterization

Around 30–40 lemons per tree were collected, and several physiological parameters were determined: fresh weight, length, and volume from the randomly selected fruits. After obtaining juice from the pulp, the pH and total sucralose content were determined by measuring the °Brix with a refractometer (Atago Co., Ltd., Bellevue, WA, USA). 

### 4.3. Pigment Analysis

Pigment analysis was carried out as proposed by Nicolas-Espinosa et al. (2022) [53]. Lemon peel from the C, M, and Z lemons was collected, and 3 mm wide pieces were trimmed to obtain 1 g of each biological sample. Then, 5 mL of methanol was added into 10 mL tubes and sealed with Parafilm. The samples were stored in darkness overnight and the pigments were measured with a spectrophotometer (Helios alpha, ThermoSpectronic, Cambridge, UK) under the following wavelengths: 450, 487.5, 502, 530, 645, and 663 nm. Pigment concentrations were calculated using Formulas (1)–(4), with A_x_ being the absorbance measured for the indicated wavelength represented. The data measured in mg·mL^−1^ were then transformed to a mg 100 g^−1^ of fresh weight.

(1) $$C_{a}\left(mg\;{mL}^{-1}\right)=\left(12.7\times A_{663}\right)-\left(2.69\times A_{645}\right)$$

(2) $$C_{b}\left(mg\;{mL}^{-1}\right)=\left(22.9\times A_{645}\right)-\left(4.68\times A_{663}\right)$$

(3) $$C_{T}\left(mg\;{mL}^{-1}\right)=\frac{0.574\times \left(4.7\times \left(A_{663}-A_{645}\right)\right)}{4.87\times \left(A_{645}-A_{633}\right)}$$

(4) $$C\left(mg\;{mL}^{-1}\right)=\left(4.07\times A_{450}\right)-\left(\left(0.0435\times C_{a}\right)-\left(0.367\times C_{b}\right)\right)$$

(5) $$L\left(\%\right)=\frac{\frac{\left(181\times A_{552}\right)}{A_{487.5}}-42}{237}\times 100$$

where Formula (1) is the chlorophyll a content (C_a_, mg mL^−1^), Formula (2) is the chlorophyll b content (C_b_, mg mL^−1^), Formula (3) is the total chlorophyll content (C_T_, mg mL^−1^), Formula (4) is the carotenoid content (C, mg mL^−1^1), and Formula (5) is the lycopene content (L, % of total carotenes).

### 4.4. Mineral Content 

The concentrations of the macro-elements (Ca, K, Mg, P, and S) and micro-elements (B, Cu, Fe, Mn, Ni, and Zn) of the pulp and peel from lemons of every treatment (C, M, and Z) were determined by inductively coupled plasma analysis (ICP). Oven-dried samples were ground (0.5–0.7 mm) with a mill grinder and digested in a microwave oven (Mars Xpress; CEM, Mattheus, NC, USA) through HNO_3_:HClO_4_ (2:1) digestion. The mineral concentration was detected by inductively coupled plasma analysis (iCAP 7000 DUO; Thermo Fisher Scientific, Waltham, MA, USA).

### 4.5. Phenolic Content Present in Fresh Lemons 

Lemon pulp and peel from the C, M, and Z lemons were separated, lyophilized, and ground. Once obtained, the phenolic compounds were extracted from the lyophilized powder (100 mg) as per Martinez-Alonso et al. (2022) [56], using a mixture of methanol:water:formic acid (25:24:1, *v*/*v*/*v*), sonicating for 1 h followed by 2–3 h of maceration at 4 °C. After centrifugation at 15,000× *g* for 15 min, the supernatants were collected and filtered through a PVDF membrane with a pore diameter of 0.22 µm. Then, the phenolic compounds were analyzed by the Experimental Science Support Service (SACE) at the University of Murcia with a HPLC-ESI-MS system consisting of an Agilent 1290 Infinity II Series HPLC (Agilent Technologies, Santa Clara, CA, USA) connected to an Agilent 6550 Q-TOF mass spectrometer (Agilent Technologies, Santa Clara, CA, USA). An Agilent Zorbax Eclipse Plus (2.1x100 mm, 1.8 um) HPLC column was used as well as MilliQ water with 0.1% formic acid as solvent A and MeOH as solvent B. 

### 4.6. Lemon-Derived Membrane Vesicles 

The pulp microsomal fraction obtention from the C, M, and Z lemons was carried out as described in Olmos-Ruiz et al. (2024) [11] with slight modification. In brief, the extraction buffer was adjusted to pH 2.5 and then the pH was set to 5.5 using Tris [(HOCH_2_)_3_CNH_2_] (Sigma-Aldrich, Darmstadt, Germany) just before the last step of extraction, which consisted of ultracentrifugation in which the microsomal fraction was obtained as a pellet. In order to determine the extraction yield, the protein concentration was determined in triplicate using the Bradford method with bovine serum albumin as the standard [57].

### 4.7. Characterization of Membrane Vesicles 

#### 4.7.1. Membrane Polydispersity and Size 

Dynamic light scattering (DLS) was used to measure, in triplicate, the particle size and polydispersity index of the microsomal fraction (0.2%) of the C, M, and Z lemons at a temperature of 20 °C using an AutoSizer-4800 spectrophotometer (Malvern Instruments, Malvern, UK). Transmission electron microscopy (TEM) was also performed to visualize the membrane vesicles, as described previously [11] (Figure S1). 

#### 4.7.2. Analysis of Membrane-Associated Phenolics and Metabolites 

In order to characterize which phenolic compounds interacted with each layer of the vesicle of the C, M, and Z membrane vesicles (500 µL), Folch extraction was carried out in triplicate by adding 750 µL of chloroform:methanol (1:2, *v*/*v*), shaking, and adding 250 µL of chloroform [58]. After this process, three different phases were obtained: an upper polar phase or fraction A, where the phenolics and other polar metabolites were retained in the solvent (methanol); an interphase or fraction B, where the proteins where compacted; and a downer non-polar phase or fraction C, where the lipids and other non-polar compounds were retained in the solvent (chloroform).

The fraction B phenolic extraction was carried out as previously described [56]. Fraction C was retained, evaporated to dryness under N_2_, and made up with 500 µL MeOH. Fraction A was polar enough to extract the phenolic compounds. The phenolic content of the three fractions was determined chromatographically as described above in triplicate.

Fraction A was also used to develop a non-directed metabolomic analysis. After drying samples with Speed-Vacuum overnight to a maximum temperature of 30 °C, they were resuspended in 100 mM potassium phosphate buffer (PB, pH 6) (diluted in 100% D2O) + 0.54 mM of TSP-d4 (internal standard). After this, the samples were filtered with 0.45 μm pore diameter Nylon filters and placed in a 5 mm NMR tube for their quantification by ^1^H-NMR.

### 4.8. Bioactivity Assays of Lemon Membrane Vesicles 

#### 4.8.1. DPPH Antioxidant Assay 

The antioxidant activity of fractions A and B of the C, M, and Z vesicles (100 µL 1:5) was determined using the 2,2-diphenyl-1-picryldhydrazyl (DPPH) radical assay as reported in Olmos-Ruiz et al. (2024) [11]. The 210 μM DPPH solution was prepared in methanol. The reaction mixtures comprising 10 μL of sample or standard (prepared with Trolox) and 190 μL of DPPH solution were added in the microplate and incubated for 30 min in the dark. The absorbance at 517 nm was measured in triplicate by using a microplate reader (PowerWave XS2, Biotek, Agilent, Santa Clara, CA, USA).

#### 4.8.2. Lipoxygenase (LOX) Inhibition Assay 

The lipoxygenase inhibition activity was assayed as reported in Tappel (1962) [59] and Ha et al. (2009) [60] with some modifications. Fraction A from the C, M, and Z vesicles was dried under N_2_ for 3 h and resuspended in 100 mM PB, pH 8, in order to perform an easier analysis. For the enzymatic assay, the incubation mixture consisted of 1.6 mL of PB pH 8, 100 µL of sample, and 100 µL of LOX enzyme solution (5000 U/mL in PB, pH 8). After incubation at room temperature for 5 min, the reaction was started by adding 200 µL of linoleic acid solution (2.5 mM in PB, pH 8), and the absorbance was measured in triplicate for 2 min at 234 nm using a spectrophotometer (Helios Zeta, Thermo Scientific). Quercetin was used as a positive control of the inhibition activity. The percentage of inhibition of lipoxygenase was calculated using Formula (6).

(6) $$Inhibition\;(\%)=\frac{E_{C}-E_{M}}{E_{C}}\times 100$$

where *E_C_* is the absorbance of the reaction without inhibitors, and *E_M_* is the absorbance of the reaction with the samples or quercetin.

### 4.9. Statistical Analysis 

Statistical analysis was performed using OriginLab (2022b) software. A Student’s *t*-test was used for the majority of analyses, except for the study of the phenolic acids associated with the plasma membrane, in which a two-way ANOVA was performed along with an HSD Tukey’s test (*p* < 0.05). All results are presented as the mean ± SEM.

## 5. Conclusions

The findings of our research indicate that the elicitation of citrus trees resulted in an enhancement in the chemical properties of the resulting lemon fruits. Initial trials utilizing ZnSO_4_ demonstrated considerable potential for elevating the phenolic compound content within the lemon pulp, thereby enhancing the antioxidant properties. Secondly, our study revealed an interaction of organic acids such as malic, ascorbic, and citric with the membrane polar components, in addition to the association with vesicles of metabolites such as phenolic acids and flavonoids, especially sinapic acid and hesperidin, and amino acids. In this way, the vesicles from the elicited material with methyl jasmonate exhibited an enhanced chemical quality of the membrane-derived nanovesicles due to an increase in the bioactive compounds associated with them, notably ascorbate and citrate. Therefore, in vitro models revealed that they were antioxidants by the scavenging of radicals and the inhibition of lipoxygenase, activities that were enhanced in vesicles derived from lemon pulp treated with methyl jasmonate. In conclusion, our findings demonstrate that methyl jasmonate treatment can provide citrus with an excellent source material for the extraction of membrane vesicles with a high content of bioactive compounds, which could be valuable for obtaining carriers of interest and applications in food, nutraceutical, and cosmetics.

## Figures and Tables

**Table 1:** Morphological characterization (weight and volume of fruit, pH, and ºBrix of juice) of the C (control), M (Methyl jasmonate treated), and Z (ZnSO_4_ treated) lemons. Data represent the mean (*n* = 15) ± SEM. The data were analyzed by a Student’s *t*-test between the treatment and the control. * *p* < 0.05.

	Fruit			Juice	
Treatment	Fresh Weight (g)	Length (cm)	Volume (cm^3^)	pH	º Brix
C (Control)	130.00 ± 3.20	8.14 ± 0.64	159.10 ± 10.1	2.23 ± 0.02	7.94 ± 0.04
M (Methyl jasmonate)	152.87 ± 4.94 *	7.85 ± 0.27	180.10 ± 11.2 *	2.23 ± 0.02	7.85 ± 0.11
Z (ZnSO_4_)	134.66 ± 5.77	8.31 ± 0.14	168.00 ± 8.13	2.18 ± 0.01	8.01 ± 0.06

**Table 2:** Morphological characterization of the C (control), M (methyl jasmonate), and Z (ZnSO_4_) vesicles. Data represent the mean ± SEM (*n* = 4). No statistical differences were observed according to a one-way ANOVA followed by HSD Tukey’s post hoc test (*p* < 0.05).

Vesicles	Protein Yield (µg µL^−1^)	Size (nm)	Polydispersity Index
C (Control)	6.65 ± 1.18	716.70 ± 139.15	0.91 ± 0.12
M (Methyl Jasmonate)	5.67 ± 1.43	654.30 ± 34.99	0.74 ± 0.14
Z (ZnSO_4_)	6.01 ± 1.13	664.45 ± 86.93	0.92 ± 0.12

**Table 3:** Metabolite content in fraction A of the C (control), M (methyl jasmonate treated), and Z (ZnSO_4_ treated) vesicles. Data represent the mean ± SEM (*n* = 4). Different letters represent significant differences according to two-way ANOVA followed by Tukey’s post hoc test (*p* < 0.05).

	Lemon-Derived Membrane Vesicles		
Metabolite (µg mg^−1^ of Protein)	C	Z	M
**GABA**	1.68 ± 0.07 c	2.13 ± 0.02 b	2.7 ± 0.05 a
**Alanine**	1.7 ± 0.07 c	2.2 ± 0.10 b	3.87 ± 0.08 a
**Asparagine**	9.52 ± 0.2 c	15.38 ± 0.17 b	25.7 ± 0.10 a
**Aspartate**	5.20 ± 0.05 c	8.1 ± 0.23 b	14.17 ± 0.26 a
**Glutamate**	2.55 ± 0.28 b	2.75 ± 0.1 b	4.5 ± 0.05 a
**Glutamine**	1.50 ± 0.02 b	1.68 ± 0.1 b	2.82 ± 0.03 a
**Isoleucine**	0.17 ± 0.1 a	0.25 ± 0.04 a	0.23 ± 0.02 a
**Leucine**	0.37 ± 0.01 a	0.36 ± 0.06 a	0.36 ± 0.2 a
**Phenylalanine**	0.30 ± 0.06 a	0.32 ± 0.03 a	0.31 ± 0.07 a
**Proline**	2.87 ± 0.02 c	4.75 ± 0.03 b	6.65 ± 0.08 a
**Threonine**	0.62 ± 0.13 a	0.68 ± 0.04 a	0.78 ± 0.1 a
**Tyrosine**	n.d.	n.d.	n.d.
**Tryptophan**	n.d.	n.d.	n.d.
**Valine**	0.27 ± 0.01 b	0.25 ± 0.02 b	0.48 ± 0.06 a
**Acetate**	0.12 ± 0.04 a	0.11 ± 0.01 a	0.09 ± 0.2 a
**Ascorbate**	11.08 ± 0.1 b	11.7 ± 0.14 b	18.00 ± 0.05 a
**Citrate**	520.4 ± 2.08 c	666.75 ± 0.7 b	851.73 ± 1.3 a
**Formate**	0.06 ± 0.00 a	0.06 ± 0.00 a	0.15 ± 0.10 a
**Fumarate**	n.d.	n.d.	n.d.
**Malate**	38.50 ± 0.2 b	47.1 ± 0.1 a	38.26 ± 0.7 b
**Succinate**	n.d.	n.d.	n.d.

## Data Availability

The data acquisition was not in legal conflict with the authorities where the work was carried out. Due to privacy issues, the individual data should not be shared. However, authors ensure that the data could be shared are in accordance with the ethical consent provided by participants on the use of confidential. The data availability will be provided upon request to the corresponding author contact information.

## Supplementary Materials

The following supporting information can be downloaded at: https://www.mdpi.com/article/10.3390/ijms252312917/s1.
