# Supplementary material for: Influence of ZnSO4 and Methyl Jasmonate on the Metabolites and Bioactivity Present in Lemon-Fruit Membrane Vesicles
Source: Int J Mol Sci. 2024 Nov 30;25(23):12917. doi: 10.3390/ijms252312917 (PMC11640879; doi:10.3390/ijms252312917)
Supplement: Supplementary file 1 [file ijms-25-12917-s001.zip › ijms-3330371-supplementary.pdf]

Supplementary Materials.

Table S1. Macronutrients and micronutrients of pulp and peel from C (control), M (methyl jasmonate treated) and Z ($ZnSO_4$ treated) lemons. Data represent the mean ± SEM (n=3). No statistical differences were observed according to a one-way ANOVA followed by HSD Tukey's post-hoc test ($p < 0.05$).

	Pulp			Peel		
Macronutrients (g 100g^{-1})	**C**	**M**	**Z**	**C**	**M**	**Z**
**Ca**	0.19±0.01	0.22±0.01	0.22±0.01	0.92±0.06	0.84±0.02	0.89±0.11
**K**	1.14±0.13	1.22±0.02	1.07±0.05	1.32±0.010	1.26±0.05	1.16±0.11
**Mg**	0.08±0.01	0.08±0.001	0.08±0.003	0.11±0.01	0.10±0.004	0.11±0.01
**P**	0.12±0.01	0.14±0.002	0.14±0.01	0.06±0.001	0.06±0.002	0.07±0.005
**S**	0.06±0.004	0.07±0.003	0.06±0.001	0.10±0.003	0.11±0.003	0.11±0.01
**Micronutrients (mg kg^{-1})**						
**B**	14.13±1.17	15.41±1.14	16.32±0.05	23.74±0.44	24.57±2.69	21.70±1.89
**Cu**	2.41±0.12	2.71±0.06	2.77±0.17	3.45±0.47	3.78±0.14	3.95±0.09
**Fe**	10.27±0.72	3.92±0.39	12.07±2.15	20.06±4.35	17.58±1.82	15.48±1.34
**Mn**	8.06±1.14	9.01±0.31	8.10±0.20	13.28±0.63	13.71±0.34	12.37±1.04
**Ni**	0.49±0.09	0.66±0.10	0.42±0.02	0.73±0.03	1.06±0.07	0.88±0.16
**Zn**	8.87±0.77	10.50±0.13	9.88±0.22	7.30±0.56	7.58±0.22	7.33±0.81

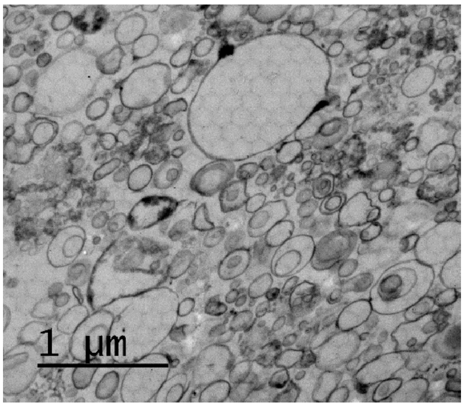

Figure S1. Image from transmission electron microscopy of lemon pulp C (Control) nanovesicles. Scale bar = 1 μm.
